# Assessment of Metabolic Dysfunction in Sepsis in a Retrospective Single-Centre Cohort

**DOI:** 10.1155/2021/3045454

**Published:** 2021-12-20

**Authors:** Julien Goutay, Juliette Perche, Aurelia Toussaint, Elodie Drumez, Michael Howsam, Claire Bourel, Benoit Brassart, Alexandre Pierre, Morgan Caplan, Arthur Durand, Marion Houard, Saad Nseir, Raphael Favory, Sébastien Preau

**Affiliations:** ^1^Division of Intensive Care, University Lille, CHU Lille, Inserm, Institut Pasteur de Lille, U1167, Lille 59000, France; ^2^Division of Intensive Care, CH Roubaix, Roubaix 59170, France; ^3^University Lille, CHU Lille, ULR 2694-METRICS: Évaluation des Technologies de Santé et des Pratiques Médicales, Lille 59000, France; ^4^University Lille, Inserm, Institut Pasteur de Lille, U1167, Lille 59000, France; ^5^Division of Intensive Care, University Lille, CHU Lille, Lille 59000, France; ^6^Division of Intensive Care, CHU Lille, Lille 59000, France

## Abstract

**Objective:**

Our primary aim was to assess selected metabolic dysfunction parameters, both independently and as a complement to the SOFA score, as predictors of short-term mortality in patients with infection admitted to the intensive care unit (ICU).

**Methods:**

We retrospectively enrolled all consecutive adult patients admitted to the eight ICUs of Lille University Hospital, between January 2015 and September 2016, with suspected or confirmed infection. We selected seven routinely measured biological and clinical parameters of metabolic dysfunction (maximal arterial lactatemia, minimal and maximal temperature, minimal and maximal glycaemia, cholesterolemia, and triglyceridemia), in addition to age and the Charlson's comorbidity score. All parameters and SOFA scores were recorded within 24 h of admission.

**Results:**

We included 956 patients with infection, among which 295 (30.9%) died within 90 days. Among the seven metabolic parameters investigated, only maximal lactatemia was associated with higher risk of 90-day hospital mortality in SOFA-adjusted analyses (SOFA-adjusted OR, 1.17; 95%CI, 1.10 to 1.25; *p* < 0.001). Age and the Charlson's comorbidity score were also statistically associated with a poor prognosis in SOFA-adjusted analyses. We were thus able to develop a metabolic failure, age, and comorbidity assessment (MACA) score based on scales of lactatemia, age, and the Charlson's score, intended for use in combination with the SOFA score.

**Conclusions:**

The maximal lactatemia level within 24 h of ICU admission is the best predictor of short-term mortality among seven measures of metabolic dysfunction. Our combined “SOFA + MACA” score could facilitate early detection of patients likely to develop severe infections. Its accuracy requires further evaluation.

## 1. Background

Sepsis is a well-recognized, worldwide healthcare issue defined as life-threatening organ dysfunction resulting from a dysregulated host response to an infection [1–3]. Septic shock is the worst form of sepsis, associated with severe hemodynamic (i.e. median arterial pressure <65 mmHg requiring vasopressor administration) and metabolic dysfunction (i.e. lactatemia ≥2 mmol/L) [4]. This condition is characterized by an abrupt and massive release of stress hormones, leading to an overwhelming production of energy substrates in the form of glucose, fatty acids, amino acids, and lactate [5]. The most severe cases tend to exhibit elevated levels of plasmatic glucose [6] and triglycerides[7], while elevated lactatemia [4, 8–12], hypothermia [13], hypoglycaemia [14, 15], and hypocholesterolemia [16–18] have all been associated with poor outcomes in critically ill patients. All these parameters are easy to measure at the bedside and may help in early detection of severe infections. However, despite its significance in the pathophysiology of both septic shock and sepsis, metabolic dysfunction only forms part of the diagnosis for the former [19, 20].

Sepsis-related organ dysfunction is characterized by an acute change of ≥2 points in the total Sequential Organ Failure Assessment (SOFA) score following infection, and this score is already in widespread clinical use [3]. However, the utility of commonly measured indicators of metabolic dysfunction as a complement to the SOFA score's prediction of short-term mortality in infected patients has yet to be studied, despite it forming part of the diagnosis of septic shock.

## 2. Methods

The primary aim of this study was to examine measurements of metabolic dysfunction, age, and the Charlson's comorbidity score (metabolic failure, age, and comorbidity assessment–MACA) as predictors of short-term mortality in patients with infection and to compare their predictive power with that of the already established SOFA score.

The secondary objective was to assess the utility of a composite score which combined the best performing of the MACA measurements with the SOFA score. We compared the relative diagnostic and prognostic performance of this combined “SOFA + MACA” score with that of the SOFA score alone, with the aim of generating a standardized, more generalized integration of metabolic dysfunction in the clinical management of cases of infection.

### 2.1. Study Design

We retrospectively enrolled all consecutive adult patients admitted to the eight intensive care units (ICUs) of the Intensive Care Centre (92 beds) in the University Hospital of Lille, between January 1^st^ 2015 and September 30^th^ 2016, with suspected or confirmed infection. A suspected infection was identified through the combination of antibiotic use (≥72 hours) and a clinician requesting body fluid culture to be performed [3]. A proven infection was defined as the invasion of normally sterile tissue, fluid, or a body cavity by pathogenic or potentially pathogenic microorganisms [21]. We considered only the first admission during the study period. We identified patients by interrogating our electronic prescription support software (IntellisPace Critical Care and Anesthesia ICCA® version H.02.01, Philips, France). This observational study was performed in strict compliance with the French reference methodology MR-004, established by the French institutional authority for personal data protection (CNIL–*Commission Nationale de l'Informatique et des Libertés*). In accordance with ethical standards, informed consent was not necessary for demographic, physiological, and hospital-outcome data analyses because this observational study did not modify existing diagnostic or therapeutic strategies. The CNIL (DEC19-259) and our institutional review board (HP20/39) approved the methodology.

### 2.2. Data Collection

Data were obtained from the electronic medical record system of the Intensive Care Centre, focusing specifically on demographic data, comorbidities, and the Charlson's score [22]. Measured physiological variables were used to calculate the SOFA score [23]. Mild-to-severe liver disease and renal insufficiency were defined respectively by a Child-Pugh score of ≥7 [24] and a creatinine clearance <60 ml/min/1.73 m^2^ of body surface [25]. Sepsis and septic shock were defined as previously described [2–4]. Community-associated infections were infections becoming manifest and being diagnosed within 48 hours of ICU admission in patients with no previous encounter with healthcare [26]. Healthcare-associated infections were defined as an infection that occurred while receiving care in a hospital or other healthcare facility, and that was neither present nor incubating upon admission [27]. Immunocompromised status was defined by the use of corticoids (>10 mg/day of prednisone or its equivalent), the administration of chemotherapy, the use of non-steroidal immunosuppressive agents following organ transplantation, or a diagnosis of autoimmune disease [28]. At hospital discharge, we collected the Rankin score [29] and the Glasgow Outcome Scale (GOS) [30].

We preselected seven biological and clinical parameters of metabolic dysfunction, all routinely measured in the participating ICUs, namely maximal arterial lactatemia, minimal and maximal temperature, minimal and maximal glycaemia, cholesterolemia, and triglyceridemia. All metabolic parameters and SOFA scores were recorded within 24 hours after ICU admission. All lactatemia measurements recorded in this study were performed on arterial blood samples using devices in the ICU (ABL 90 Flex, Radiometer TM [31]). Age and the Charlson's comorbidity score were integrated with these 7 measurements of metabolic dysfunction to generate a new score representing MACA.

### 2.3. Statistical Analysis

Quantitative variables were expressed as medians (interquartile range, IQR). Normality of distributions was assessed using histograms and the Shapiro-Wilk test. Categorical variables were expressed as numbers (percentages). The magnitude of differences between groups according to 90-day mortality status was assessed using absolute standardized differences (ASD), with an ASD >20% being interpreted as a meaningful difference. Because the present study additionally aimed to assess the utility of metabolic dysfunction parameters as a complement to the SOFA score, we specifically focused our analyses on metabolic parameters for which daily measurements were readily available. To account for two important predictors of mortality, age and the Charlson's comorbidity score were also included as candidate predictors of interest into the “MACA” score. We compared the MACA score between survivors and non-survivors (in-hospital mortality within 90 days) using unadjusted and SOFA-adjusted logistic regression models. For each parameter, the assumption of log-linearity for identical SOFA scores was verified using the cubic splines function. For parameter(s) significantly associated with in-hospital mortality within 90 days in SOFA-adjusted analysis (i.e. parameter(s) of interest), we developed a scoring-point system using the regression coefficients of multivariable logistic regression models, dividing the values of the selected parameter(s) into 5 levels and using the Framingham risk development method [32]. To assess this new MACA scoring system, ultimately intended for use in combination with the SOFA score, we estimated the selected parameters' coefficients by reflecting the increase in mortality risk per 1 point of the SOFA score. Parameter intervals were expressed as [a-b), indicating an interval from a to *b*, that is, inclusive of a, but exclusive of *b*. Calibration of this combined “SOFA + MACA” model was assessed by the Hosmer and Lemeshow test [33] and by plotting the observed probability against the predicted probability in deciles. We evaluated the prognostic performance of the combined “SOFA + MACA” model to predict 90-day mortality by calculating the area under (AUC) the receiver operating characteristic (ROC) curve and using the De Long test to compare with the prognostic performance of a model using only the SOFA score. Statistical testing was performed at the two-tailed *α* level of 0.05. Data were analyzed using the SAS software package, release 9.4 (SAS Institute, Cary, NC).

## 3. Results

**Figure 1:**
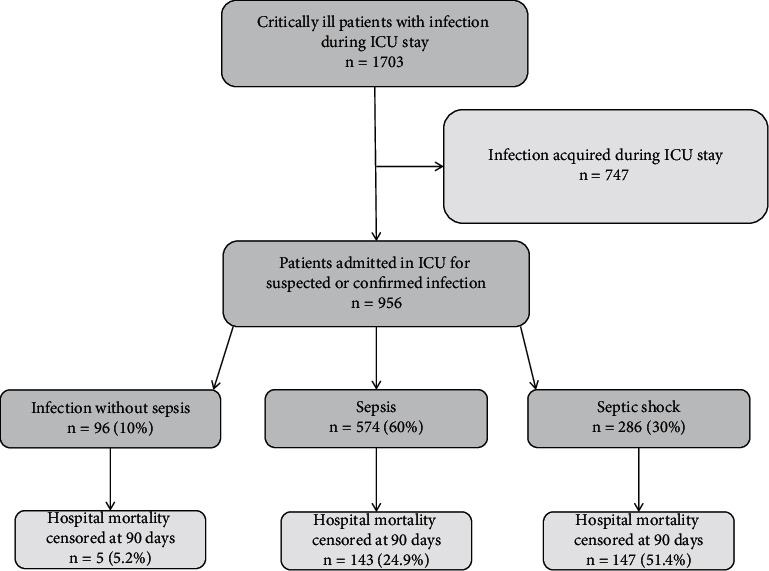
Number of patients who were screened and finally included in the analysis. ICU, intensive care unit.

From January 1^st^ 2015 to September 30^th^ 2016, 956 patients were admitted to ICU for infection (see Figure 1). The patients were mostly male (*n* = 600, 62.7%), and the median age was 63 [34, 35] years (see Table 1). Within the first 24 hours in ICU, all the patients received both antibiotics and blood volume expansion, 796 patients (83.3%) received at least 30 mL/Kg of vascular filling, 435 (45.5%) required vasopressors and 413 (43.2%) were intubated. Characteristics of the initial infection are detailed in supplemental file 1: infections were mostly community acquired (*n* = 519, 54.3%) and were *pneumoniae* for 473 (49.5%) patients, necrotizing dermohypodermitis for 152 (15.9%), and acute pyelonephritis for 100 (10.5%). The microbiological sampling was positive in 553 cases (57.8%); the vast majority of which indicated a bacterial source of infection (*n* = 496, 51.9%). Two hundred and ninety-five (30.9%) patients died within the first 90 days following admission to intensive care.

Missing metabolic parameters were as follows: no patients lacked data for body temperature, 1 (0.1%) lacked data for glycaemia and 36 (3.8%) for lactatemia, and 151 (15.8%) for both cholesterolemia and triglyceridemia. Maximal lactatemia level within 24 hours of admission to ICU was the only metabolic parameter associated with a higher risk of 90-day hospital mortality in both unadjusted (odds ratio (OR), 1.31; 95%CI, 1.23 to 1.40; *p* < 0.001) and SOFA-adjusted (SOFA-adjusted OR, 1.17; 95%CI, 1.10 to 1.25; *p* < 0.001; Table 2) analyses. Age and Charlson's comorbidity score were the only two nonmetabolic parameters with an ASD >25%. They remained significantly associated with 90-day mortality in both unadjusted and SOFA-adjusted analyses (Table 2).

**Figure 2:**
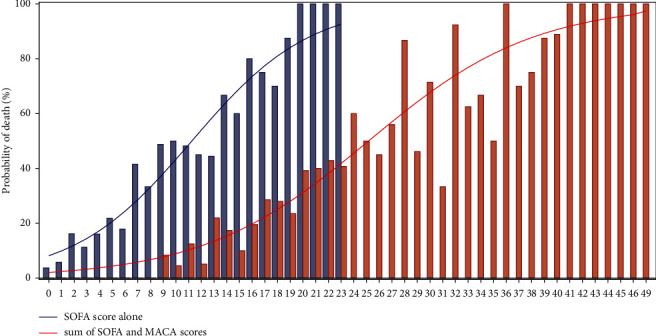
Predicted probability of in-hospital mortality within 90-days of intensive care admission for severity scores. Histograms represents observed probability of death and lines represents predicted probability of death. SOFA, Sequential Organ Failure Assessment; MACA, metabolic failure, age, and comorbidity assessment. No patient had a total SOFA score of 24 points in our study. No patient had a total combined “SOFA + MACA” model score of 50 points or more.

**Figure 3:**
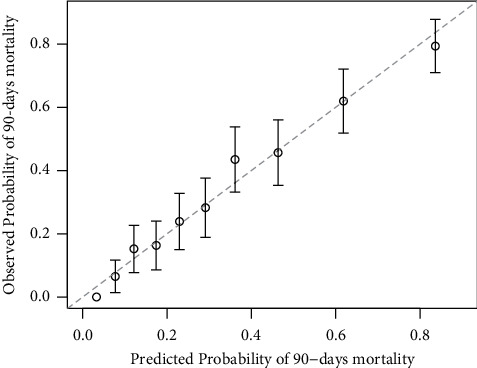
Calibration plot of the combined “SOFA + MACA” model to predict short-term mortality. Regarding the Hosmer and Lemeshow test, this combined “SOFA + MACA” model exhibited a good calibration (*p*=0.47). MACA, metabolic failure, age, and comorbidity assessment; SOFA, sequential organ failure assessment.

Table 3 presents the MACA model while Figure 2 presents the predicted probabilities of death for each point of the SOFA score and each point of the new model resulting from the sum of the SOFA and MACA scores. Regarding the Hosmer and Lemeshow test, the combined “SOFA + MACA” model exhibited a good calibration (Figure 3, *p*=0.47). The distribution of patients' SOFA score alone and the combined “SOFA + MACA” model according to infection severity is presented in Supplemental file 2.

The performance of the combined “SOFA + MACA” model to predict 90-day hospital mortality (AUC, 0.81; 95%CI, 0.78 to 0.84) was significantly better than the model based solely upon the SOFA score (AUC 0.74; 95%CI, 0.71 to 0.77; *p* < 0.001).

## 4. Discussion

We report here the first study to assess an integrated measure of sepsis-related metabolic dysfunction integrating seven commonly available clinical and biological markers, age, and the Charlson's comorbidity score (MACA). Among the preselected candidates of metabolic dysfunction, only maximal lactatemia level within the first 24h of ICU admission was associated with a higher risk of mortality in the SOFA-adjusted analysis, while the two background characteristics (age and Charlson's comorbidity score) were also associated with a higher risk of mortality in SOFA-adjusted analyses. The performance of a combined “SOFA + MACA” model to predict 90-day hospital mortality was better than the model based solely upon the SOFA score.

The present study is the first to describe a multivariate analysis comparing the relative association between several metabolic parameters and mortality. Previous studies already showed the prognostic value of hypothermia [13], hypoglycaemia [14, 15], hypocholesterolemia [16–18], and hyperlactatemia [9, 10, 36–44], but none performed a global analysis of their respective impact on mortality. High lactate levels are commonly associated with poor outcome in critically ill patients, including those admitted for sepsis or septic shock [9, 10, 36–44]. Howell et al. have also demonstrated that hyperlactatemia is an independent risk factor of 28-day mortality in a prospective cohort of 1287 patients admitted to the emergency department with an infection [45]. Thus, assessment of plasmatic lactate has already been proposed for risk stratification of patients admitted to the hospital for infection or sepsis [12, 45]. Some authors have shown the utility of combining lactatemia with the quick-SOFA score (qSOFA) in non-ICU patients [46, 47]. Our results further suggest that in combination with the SOFA score, lactatemia measurements may be helpful in deciding which patients could benefit from more proactive treatment strategies and early admission to ICU. First, the present study suggests that patients with isolated lactatemia ≥2 mmol/L, or a combination of a lactatemia of [1.0–2.0) mmol/L and a total SOFA score of 1, might be considered as having a cryptic sepsis and should be treated accordingly [48, 49]. Other studies have also reported that lactatemia levels ostensibly in the “normal range” (i.e. between 1 and 2 mmol/L) are associated with short- and long-term mortality in patients with sepsis [4, 50, 51].

In patients with elevated lactatemia upon admission, previous studies have already shown the advantages of dynamic lactate measurements to predict hospital mortality (namely the “lactime” concept developed by Bakker et al. [52], time-adjusted lactatemia [53], lactate decrease [34, 54, 55], or lactate normalization [56]), and it has been recommended that normalization of lactatemia may be used as an early therapeutic target in patients with sepsis or septic shock [31, 49, 57]. We nevertheless chose to study the static maximal lactatemia within the first 24h of ICU admission, primarily to facilitate its inclusion to a score that could be ultimately combined with the total SOFA score [23].

Our study also found an association between both the plasmatic cholesterol level and the minimal body temperature with short-term mortality in unadjusted analyses. As previously reported, patients with a lower cholesterol level on admission had a higher risk of mortality [18, 58]. Cholesterol's role in bacterial endotoxin neutralization [59], its impacts on cellular immunity [60], its role in steroid biosynthesis [61], or indeed a combination of these mechanisms may also explain these findings. Moreover, and also as previously published, nonsurvivors were more likely to have a lower temperature on admission [13]. This may be the reflection of the sick euthyroid syndrome observed in the first phase of hormonal impairment [62]. Nonetheless, in contrast to lactatemia, plasmatic cholesterol levels and body temperature were not associated with mortality in SOFA-adjusted analyses. The multimodal mechanisms leading to hyperlactatemia in infected patients may be responsible for its particular relevance for short-term outcomes and make it a good candidate to complement the classical assessment of severe infections using the SOFA score. Indeed, rather than a simple reflection of an impaired oxygen delivery leading to an anaerobic metabolism [63, 64], lactate production is a sensitive, standalone indicator of a wide range of metabolic impairments at the cellular level. As observed by Alvarez-Garcia *et al.*, the link between tissue hypoxia and lactate production in humans with severe infection remains to be demonstrated [65], but lactatemia is essentially the result of an imbalance between lactate production and elimination. On the one hand, the main mechanisms thought to be responsible for an increase in aerobic lactate production during sepsis are an increase in aerobic glycolysis, an inhibition of the pyruvate dehydrogenase, and dysfunction of the mitochondrial electron transport chain [66–68]. On the other hand, the main metabolic alterations proposed as being responsible for reduced lactate extraction from the blood during sepsis are a gluconeogenesis insufficiency in the liver and kidney and a lack of lactate oxidation in the heart and brain [66–68].

It is interesting to note that maximal lactatemia remained significantly associated with a poor prognosis, even if age and the Charlson's score were taken into account. We identified accurate, modifiable (i.e. maximal lactatemia), and nonmodifiable (i.e. age and comorbidities) factors which predict mortality in combination with the SOFA score in patients hospitalized for a restricted list of potentially severe infections (*pneumoniae*, necrotizing dermohypodermitis, acute pyelonephritis, acute peritonitis, endocarditis, central venous catheter infection, meningitis, and acute osteoarthritis). Recently, other authors have shown the predictive value of age for sepsis-related mortality [69–71], but none demonstrated an equivalence between “modifiable” and “non-modifiable” factors using a scoring-point system of mortality prediction. Thus, it is interesting that in terms of mortality prediction and in this specific population, a lactatemia of ≥2 mmol/L is barely equivalent to an age of ≥40 years or a Charlson's comorbidity score of ≥2 (Table 3). Moreover, our results showed that patients hospitalized for *pneumoniae*, necrotizing dermohypodermitis, acute pyelonephritis, acute peritonitis, endocarditis, central venous catheter infection, meningitis, or acute osteoarthritis, with an age ≥40 years or a Charlson's score ≥2, might also be considered as having a cryptic sepsis and should be treated accordingly [48, 49].

We are aware of the limitations of our study. First, the single-centre design may limit the external validity of the results. Nevertheless, we observed similar demographic data to previously studied populations [4, 35, 72]. Chronic pulmonary disease, diabetes, and chronic heart and renal failure were the most frequent coexisting disorders [35]. Concerning hospital mortality, we here report a higher mortality rate (31.5%) than reported by Raith et al. (18.7%) [72], but one which is similar to that reported in a British cohort [73] or in the ANDROMEDA-SHOCK trial [74]. The severity on admission was similar to other published data, regarding both the SOFA scores [4, 72] and the proportion of infection without sepsis or septic shock [72, 73]. The lower respiratory tract was also the most common infection site [54, 72, 73], while infection of skin and soft tissues was the second-most common origin of sepsis. This finding could well be due to a local particularity in admissions at the Lille University Hospital, serving as it does as a referral centre for patients with skin and soft tissue infections. Second, our results were obtained from a population of patients admitted to ICU with potentially severe infections (*pneumoniae*, necrotizing dermohypodermitis, acute pyelonephritis, acute peritonitis, endocarditis, central venous catheter infection, meningitis, and acute osteoarthritis). Thus, they need to be confirmed in further studies before they can be generalized to an unselected population of patients with less-specific suspected or known infection. Third, as it is impossible to determine the precise cause of hyperlactatemia in the context of infection, we did not account for the potential for treatment-related hyperlactatemia in the analyses of our results, such as metformin overdose [75] or *β*2 mimetic administration [76]. Finally, lactatemia measurements were performed with ICU devices as part of local management protocols [77], primarily to avoid mismeasurement due to the time of transport to the laboratory: our results therefore require confirmation with laboratory-based analytical devices for lactatemia measurements but are nevertheless representative of a real-world clinical situation.

## 5. Conclusion

Metabolic dysfunction is a major component of the pathophysiology of severe infections but nevertheless only forms part of the diagnosis of septic shock, not sepsis. Maximal lactatemia within the first 24 hours in ICU, as an adjunct to the SOFA score, is the best predictor of short-term mortality from among seven measurements of metabolic function commonly available in clinical practice. We also developed a new metabolic failure, age, and comorbidity assessment (MACA) score that included this measure of lactatemia, the patient's age, and Charlson's comorbidity score. Used in combination with the SOFA score, this MACA score could help physicians to diagnose cases of cryptic sepsis and may thus be helpful in deciding which patients could benefit from more proactive treatment strategies and early admission to ICU. The accuracy of this new MACA score needs to be evaluated in future studies, but it has the merit of including physiologically relevant, routine measurements to augment an already well-established risk assessment tool.

## Figures and Tables

**Table 1:** Main characteristics of hospital survivors and nonsurvivors to infections at 90 days.

	In-hospital survivors within 90 days (*n* = 661)	In-hospital nonsurvivorsg within 90 days (*n* = 295)	ASD (%)
Age (years)^∗∗^	60 (50, 70)	66 (58, 77)	45.6
Male sex, *n* (%)^∗∗^	419 (63.4)	181 (61.4)	4.2
Weight (kg)^∗∗^	76 (65; 91)	75 (65; 90)	6.5
Medical history^∗∗^			
Chronic pulmonary disease	192 (29.0)	88 (29.8)	1.7
Diabetes	149 (22.5)	71 (24.1)	0.5
Chronic heart failure	102 (15.4)	69 (23.4)	20.2
Coronary arterial disease	86 (13.0)	61 (20.7)	20.6
Peripheral arterial disease	64 (9.7)	38 (12.9)	10.1
Stroke	90 (13.6)	60 (20.3)	12.7
Cognitive disorder	22 (3.3)	19 (6.4)	14.5
Diffuse connect tissue disease	38 (5.7)	27 (9.2)	13.0
Peptic ulcer disease	11 (1.7)	10 (3.4)	11.0
Liver disease	42 (6.4)	37 (12.5)	21.3
Chronic renal failure (mild to severe)	*46 (7.0)*	*34 (11.5)*	*15.8*
Solid cancer	142 (21.5)	86 (29.2)	17.7
Leukemia	48 (7.3)	36 (12.2)	16.7
Lymphoma	24 (3.6)	23 (7.8)	18.0
HIV infection	1 (0.1)	2 (0.7)	8.2
Immunocompromised	135 (20.4)	90 (30.1)	23.3
Charlson's comorbidity score	2 [1–4]	3 [2–6]	60.5
Baseline SOFA	0 [0–0]	0 [0–0]	2.9
Severity scores and metabolic parameters within 24 hours after ICU admission			
SOFA^∗∗^	5 (3, 8)	9 (6, 13)	96.5
Maximal lactatemia, (mmol/L)^*∗*^	1.6 (1.0; 2.6)	2.9 (1.5; 5.1)	69.9
Minimal glycaemia (g/L)^*∗*^	1.1 (0.9; 1.3)	1.0 (0.8; 1.3)	15.1
Maximal glycaemia (g/L)^*∗*^	1.6 (1.3; 2.1)	1.6 (1.2; 2.2)	4.1
Minimal temperature (°C)^*∗*^	36.5 (36.0; 37.1)	36.4 (35.8; 37.1)	12.5
Maximal temperature (°C)^*∗*^	37.9 (37.2; 38.6)	37.8 (36.8; 38.7)	9.8
Total serum cholesterol (g/L)^*∗*^	1.1 (0.8; 1.4)	0.9 (0.7; 1.3)	30.3
Total serum triglycerides (g/L)^*∗*^	1.1 (0.8; 1.7)	1.1 (0.8; 1.6)	5.5
Characteristics at hospital discharge^∗∗^			
ICU LOS, days	8 (4, 16)	5 (2, 14)	—
Hospital LOS, days	16 (8, 29)	7 (2, 19)	—
Glasgow outcome scale	4 (4, 5)	1	—
Rankin score	1 (0; 2)	6	—

Values are expressed as counts (%) or medians (25^th^; 75^th^ centiles). ASD, absolute standardized difference, ICU, intensive care unit; LOS, length of stay; SOFA : sequential organ failure Assessment. ^*∗*^Missing metabolic parameters were as follows: no patients lacked data for body temperature, 1 (0.1%) lacked data for glycaemia, 36 (3.8%) for lactatemia, and 151 (15.8%) for both cholesterolemia and triglyceridemia. ^∗∗^ No missing data.

**Table 2:** Unadjusted and SOFA-adjusted odds ratio for in-hospital mortality within 90 days of intensive care admission.

Variable within the first 24 hours	Unadjusted analyses		SOFA-adjusted analyses	
	OR [95%CI]	*p* value	OR [95%CI]	*p* value
Maximal lactatemia^*∗*^	1.31 [1.23; 1.40]	**<0.001**	1.17 [1.10; 1.25]	**<0.001**
Minimal glycaemia^*∗*^	0.85 [0.61; 1.17]	0.31	0.84 [0.60; 1.18]	0.32
Maximal glycaemia^*∗*^	0.95 [0.80; 1.13]	0.59	0.85 [0.70; 1.03]	0.089
Minimal temperature^*∗*^	0.85 [0.75; 0.96]	**0.009**	0.95 [0.83; 1.08]	0.41
Maximal temperature^*∗*^	0.91 [0.80; 1.03]	0.12	0.89 [0.77; 1.01]	0.076
Cholesterol level^*∗*^	0.57 [0.41; 0.80]	**0.001**	0.81 [0.59; 1.13]	0.21
Triglyceride level^*∗*^	1.02 [0.89; 1.16]	0.83	0.90 [0.77; 1.05]	0.16
Age^*∗*^	1.03 [1.02; 1.04]	**<0.001**	1.04 [1.03; 1.05]	**<0.001**
Charlson's comorbidity score^*∗*^	1.22 [1.16; 1.28]	**<0.001**	1.24 [1.17; 1.32]	**<0.001**

CI, confidence interval; OR: odds ratio; SOFA, sequential organ failure assessment. ^*∗*^Missing parameters were as follows: no patients lacked data for body temperature, age, and Charlson's comorbidity score, 1 (0.1%) lacked data for glycaemia, 36 (3.8%) for lactatemia, and 151 (15.8%) for both cholesterolemia and triglyceridemia Bold values highlight the significant *p* values.

**Table 3:** The metabolic failure, age, and comorbidity assessment (MACA) score.

Parameters	Categories				
Maximal lactatemia (mmol/L)	<1	[1.0–2.0)	[2.0–4.0)	[4.0–6.0)	≥6
Number of patients	149	340	256	84	91
Regression coefficients^*∗*^	(Ref)	0.74	2.03	3.76	13.3
Attributed points	0	1	2	4	13
Age	<40	[40–55)	[55–65)	[65–80)	≥80
Number of patients	102	177	227	333	117
Regression coefficients^*∗*^	(Ref)	3.70	6.02	8.34	11.40
Attributed points	0	4	6	8	11
Charlson's comorbidity score	0–1	2–3	4–5	6–7	≥8
Number of patients	318	337	147	60	94
Regression coefficients^*∗*^	(Ref)	2.08	4.17	6.25	10.94
Attributed points	0	2	4	6	11

The metabolic failure, age and comorbidity assessment (MACA) score according to maximal lactatemia within the first 24 hours of intensive care admission and to background characteristics (i.e: age and Charlson's comorbidity score). ^*∗*^Regression coefficients associated with maximal lactatemia, age, or Charlson's score calculated using the Framingham method to reflect the increase associated with one point of the sequential organ failure assessment (SOFA) score. The notation [a-b) indicates an interval from a to *b*, that, is inclusive of a but exclusive of *b*.

## Data Availability

The data are available on reasonable request sent to the corresponding author.

## Abbreviations

AUC: Area under the curve
BAL: Bronchoalveolar lavage
ICU: Intensive care unit
IQR: Interquartile range
IV: Intravenous
GOS: Glasgow outcome scale
MACA: Metabolic failure, age and comorbidity assessment
OR: Odds ratio
qSOFA: Quick sequential organ failure assessment
ROC: Receiver operating characteristic
SOFA: Sequential organ failure assessment
UCBE: Urine cytobacteriological examination.

## Supplementary Materials

Supplementary Materials Supplemental file 1: Characteristics of infections leading to intensive care admission among hospital survivors and non-survivors. Values are expressed as counts (%) or medians (25th; 75th centiles). BAL, bronchoalveolar lavage; UCBE, urine cytobacteriological examination. Supplemental file 2. Distribution of the SOFA and MACA scores according to infection severity. SOFA, Sequential Organ Failure Assessment; MACA, Metabolic failure, age and comorbidity assessment. Infection, sepsis and septic shock are defined according to SEPSIS-3 definitions [1]. 1. Singer *M*, Deutschman CS, Seymour CW, Shankar-Hari *M*, Annane D, Bauer *M*, et al. The Third International Consensus definitions for Sepsis and Septic Shock (Sepsis-3). JAMA. 2016; 315 : 801–10 .
