# Supplementary material for: Assessment of Metabolic Dysfunction in Sepsis in a Retrospective Single-Centre Cohort
Source: Crit Care Res Pract. 2021 Dec 20;2021:3045454. doi: 10.1155/2021/3045454 (PMC8712182; doi:10.1155/2021/3045454)
Supplement: Supplementary Materials — Supplemental file 1: Characteristics of infections leading to intensive care admission among hospital survivors and non-survivors. Values are expressed as counts (%) or medians (25th; 75th centiles). BAL, bronchoalveolar lavage; UCBE, urine cytobacteriological examination. Supplemental file 2. Distribution of the SOFA and MACA scores according to infection severity. SOFA, Sequential Organ Failure Assessment; MACA, Metabolic failure, age and comorbidity assessment. Infection, sepsis and septic shock are defined according to SEPSIS-3 definitions [1]. 1. Singer M, Deutschman CS, Seymour CW, Shankar-Hari M, Annane D, Bauer M, et al. The Third International Consensus definitions for Sepsis and Septic Shock (Sepsis-3). JAMA. 2016; 315 : 801–10 . [file 3045454.f1.zip › 3045454.f1/Supplemental file 1 (1).docx]

**Supplemental file 1: Characteristics of infections leading to intensive care admission among hospital survivors and non-survivors.**

|  | **In-hospital survivors within 90 days**  **(n = 661)** | **In-hospital non-survivors within 90 days**  **(n = 295)** |
| --- | --- | --- |
| Community acquired | 393 (59.5) | 126 (42.7) |
| Health care associated | 268 (40.5) | 169 (57.3) |
| Pneumoniae | 308 (46.6) | 165 (55.9) |
| Necrotizing dermohypodermitis | 125 (18.9) | 27 (9.1) |
| Acute pyelonephritis | 73 (11.0) | 27 (9.1) |
| Acute peritonitis | 57 (8.6) | 38 (12.9) |
| Endocarditis | 13 (2.0) | 14 (4.7) |
| Central venous catheter | 17 (2.6) | 8 (2.7) |
| Meningitis | 13 (2.0) | 1 (0.3) |
| Acute osteoarthritis | 9 (1.4) | 0 (0.0) |
| Others | 46 (7.0) | 15 (5.1) |
| No identified pathogens | 276 (41.8) | 127 (43.1) |
| Identified pathogens | 385 (58.2) | 168 (56.9) |
| *Sputum or tracheal aspiration* | *83 (12.6)* | *35 (11.9)* |
| *BAL* | *24 (3.6)* | *25 (8.5)* |
| *UCBE* | *58 (8.8)* | *19 (6.4)* |
| *Blood culture* | *139 (21.0)* | *80 (2.7)* |
| *Urinary antigens* | *22 (3.3)* | *11 (3.7)* |
| *Lombar punction* | *8 (1.2)* | *1 (0.3)* |
| *Per operative sampling* | *83 (12.6)* | *18 (6.1)* |
| *Nasal swab* | *27 (4.0)* | *9 (3.1)* |
| *Stool culture* | *4 (0.6)* | *1 (0.3)* |
| Bacteria | 353 (53.4) | 143 (48.5) |
| Virus | 37 (5.6) | 15 (5.1) |
| Parasite | 2 (0.3) | 2 (0.7) |
| Fungi | 11 (1.7) | 24 (8.1) |
| Underwent surgery, n (%) | 200 (30.3) | 54 (18.3) |
| *Time from admission to surgery, days* | *0 (0; 0)* | *0 (0; 0)* |

*Values are expressed as counts (%) or medians (25^th^; 75^th^ centiles). BAL, bronchoalveolar lavage; UCBE, urine cytobacteriological examination*
