# Supplementary material for: Assessment of Metabolic Dysfunction in Sepsis in a Retrospective Single-Centre Cohort
Source: Crit Care Res Pract. 2021 Dec 20;2021:3045454. doi: 10.1155/2021/3045454 (PMC8712182; doi:10.1155/2021/3045454)
Supplement: Supplementary Materials — Supplemental file 1: Characteristics of infections leading to intensive care admission among hospital survivors and non-survivors. Values are expressed as counts (%) or medians (25th; 75th centiles). BAL, bronchoalveolar lavage; UCBE, urine cytobacteriological examination. Supplemental file 2. Distribution of the SOFA and MACA scores according to infection severity. SOFA, Sequential Organ Failure Assessment; MACA, Metabolic failure, age and comorbidity assessment. Infection, sepsis and septic shock are defined according to SEPSIS-3 definitions [1]. 1. Singer M, Deutschman CS, Seymour CW, Shankar-Hari M, Annane D, Bauer M, et al. The Third International Consensus definitions for Sepsis and Septic Shock (Sepsis-3). JAMA. 2016; 315 : 801–10 . [file 3045454.f1.zip › 3045454.f1/Supplemental file 2 (figure + legend).docx]

**Supplemental file 2. Distribution of the organ and metabolism dysfunction scores according to infection severity.**

SOFA, Sequential Organ Failure Assessment; MACA, Metabolic failure, age and Charlson’s comorbidity assessment. Infection, sepsis and septic shock are defined according to SEPSIS-3 definitions (2).


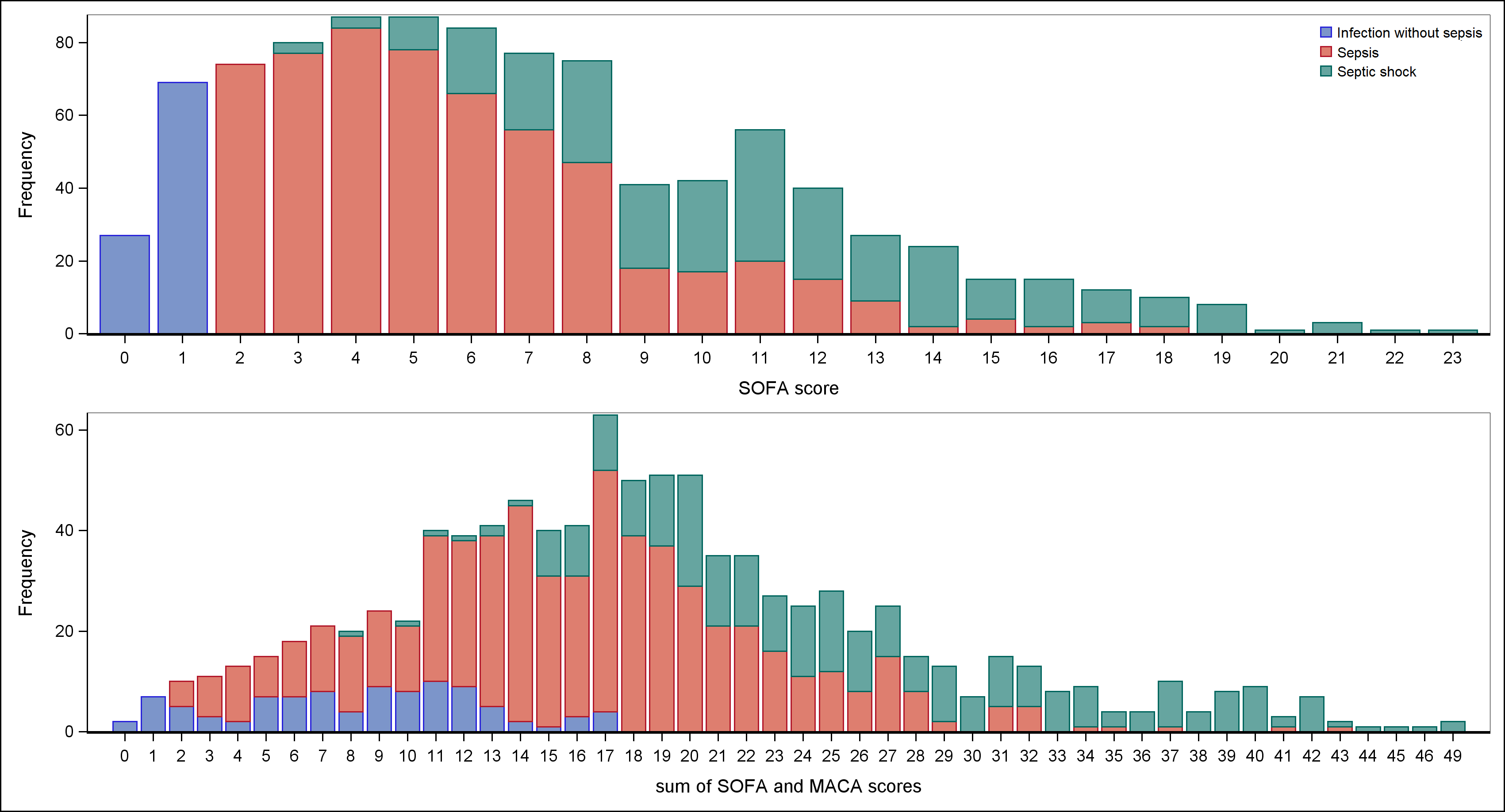


1. Singer M, Deutschman CS, Seymour CW, Shankar-Hari M, Annane D, Bauer M, et al. The Third International Consensus Definitions for Sepsis and Septic Shock (Sepsis-3). JAMA. 2016;315:801–10.
